# Scaling Analysis of Ocean Surface Turbulent Heterogeneities from Satellite Remote Sensing: Use of 2D Structure Functions

**DOI:** 10.1371/journal.pone.0126975

**Published:** 2015-05-27

**Authors:** P. R. Renosh, Francois G. Schmitt, Hubert Loisel

**Affiliations:** 1 University of Lille, Laboratory of Oceanology and Geosciences, UMR 8187 LOG CNRS/Univ Lille/ULCO, Wimereux, France; 2 CNRS, Laboratory of Oceanology and Geosciences, UMR 8187 LOG CNRS/Univ Lille/ULCO, Wimereux, France; 3 ULCO, Laboratory of Oceanology and Geosciences, UMR 8187 LOG CNRS/Univ Lille/ULCO, Wimereux, France; University of California San Diego, UNITED STATES

## Abstract

Satellite remote sensing observations allow the ocean surface to be sampled synoptically over large spatio-temporal scales. The images provided from visible and thermal infrared satellite observations are widely used in physical, biological, and ecological oceanography. The present work proposes a method to understand the multi-scaling properties of satellite products such as the Chlorophyll-a (Chl-a), and the Sea Surface Temperature (SST), rarely studied. The specific objectives of this study are to show how the small scale heterogeneities of satellite images can be characterised using tools borrowed from the fields of turbulence. For that purpose, we show how the structure function, which is classically used in the frame of scaling time series analysis, can be used also in 2D. The main advantage of this method is that it can be applied to process images which have missing data. Based on both simulated and real images, we demonstrate that coarse-graining (CG) of a gradient modulus transform of the original image does not provide correct scaling exponents. We show, using a fractional Brownian simulation in 2D, that the structure function (SF) can be used with randomly sampled couple of points, and verify that 1 million of couple of points provides enough statistics.

## Introduction

One of the main features of geophysical fields is their huge fluctuations occurring over wide ranges of spatio-temporal scales. Here we consider the heterogeneities and intermittencies in 3D ocean turbulence. We use for this the framework of homogeneous and locally isotropic turbulence that originated in the work of Kolmogorov [1]. In this framework, energy is supplied, introduced or produced in the fluid at a relatively large scale, and is successively passed by interactions between eddies or their instabilities. This is performed through a spectrum of smaller and smaller eddies where inertial forces are dominant. After successive cascades steps, these eddies are conveyed to eddies of size comparable to the Kolmogorov length scale *η*, where viscosity plays a major role in transferring their kinetic energy into heat. This was formalised using the velocity fluctuations at scale *l*; for time series it writes Δ*V*_*l*_ = ∣*V*(*x* + *l*) − *V*(*x*)∣ (*V* is the velocity); and for an isotropic 2D field it can be written Δ*V*_*l*_ = ∥ *V*(*M*) − *V*(*N*) ∥, where *M* and *N* are two points and *l* = *d*(*M*,*N*):

$$\begin{array}{c} \left\langle \Delta V_{l}\right\rangle =C\epsilon^{1/3}l^{1/3} \end{array}$$ (1)

where ⟨⟩ means statistical average, *C* is a constant and *ϵ* represents the dissipation. This can also be written in the spectral space as follows [2]:

$$\begin{array}{c} E_{v}\left(k\right)=C_{1}\epsilon^{2/3}k^{-5/3} \end{array}$$ (2)

where *C*_1_ is another constant, *E*_*v*_(*k*) is the Fourier spectral energy of velocity, and *k* is the wave number. This corresponds to a situation of scale invariance: velocity fluctuation have no characteristic scale with a power-law scale dependence. A similar scale dependence can be obtained for a passive scalar *θ*, with a power-law of the form [3, 4]:

$$\begin{array}{c} E_{\theta }\left(k\right)=C_{2}\epsilon^{-1/3}\chi k^{-5/3} \end{array}$$ (3)

where *E*_*θ*_(*k*) is the Fourier spectral energy of passive scalar, *C*_2_ is another constant, and *χ* is the dissipation of scalar variance (analogous to *ϵ* as dissipation of kinetic energy). It is now realized for a long time that turbulence produces intermittency, i.e. huge local fluctuations in energy and passive scalar fluxes *ϵ* and *χ*, and large variations in velocity and passive scalars [5]. Since the proposals of Obukhov and Kolmogorov in 1962 [6, 7] those quantities are characterized using local averages *ϵ*_*l*_ and *χ*_*l*_ at scale *l*:

$$\begin{array}{c} \epsilon_{l}\left(x\right)=\frac{1}{a_{l}}\int_{B_{l}\left(x\right)}\epsilon \left(x^{\prime }\right)dx^{\prime };\;\chi_{l}\left(x\right)=\frac{1}{a_{l}}\int_{B_{l}\left(x\right)}\chi \left(x^{\prime }\right)dx^{\prime } \end{array}$$ (4)

where *B*_*l*_(*x*) is a bowl of radius *l* centered in *x* and $a_{l}=\frac{4}{3}\pi l^{3}$ is its volume. This is called the “coarse graining”method (CG). This method is used to change the resolution of a positive, intermittent field. These local averages have scaling statistical properties of the form [5, 8]:

$$\begin{array}{c} \left\langle \epsilon_{l}^{q}\right\rangle \approx l^{-K_{\epsilon }\left(q\right)};\;\left\langle \chi_{l}^{q}\right\rangle \approx l^{-K_{\chi }\left(q\right)} \end{array}$$ (5)

where *q* is the statistical moment, *K*_*ϵ*_(*q*) and *K*_*χ*_(*q*) are scale invariant moment functions; these are also second Laplace characteristic function and as such are convex functions. They verify *K*_*ϵ*_(1) = 0 and *K*_*χ*_(1) = 0 by conservation of fluxes. Another approach to characterize intermittency and local fluctuations in the studied fields is to directly characterize the fluctuations of velocity and passive scalar using structure functions [5]:

$$\begin{array}{c} \left\langle \Delta V_{l}^{q}\right\rangle \approx l^{\zeta_{v}\left(q\right)};\;\left\langle \Delta \theta_{l}^{q}\right\rangle \approx l^{\zeta_{\theta }\left(q\right)} \end{array}$$ (6)

where *ζ*_*v*_(*q*) and *ζ*_*θ*_(*q*) are the scaling moment functions that characterize the fluctuations of velocity and passive scalar [9]. This is called the structure function method (SF). In the following, we focus on the passive scalar case, since we will consider Chlorophyll-a and Sea Surface Temperature, which are transported scalars and may be compared to passive scalars. The scaling moment functions for both CG and SF methods are derived using remotely sensed 2D Chl-a and SST images from MODIS Aqua.

In the next section we present the two-dimensional data analysis techniques using CG and SF methods. The next section deals with the test of these two methods for various 2D stochastic simulations. Finally as an illustration, the methods are applied to two real images (Chl-a and SST) measured from MODIS Aqua. An often assumed link between scaling exponents estimated using CG and SF methods is tested on these images and shown to be wrong except for low order moments.

## Methods

### Data analysis techniques

Multifractal methods have been widely applied to time series, but there are not many studies applying such approaches to 2D data, especially in the field of ocean color remote sensing. Some of them considered a local gradient transform in order to identify currents and oil spills [10–13]. Other studies transformed satellite Chl-a or SST image data into a positive singular field using a gradient modulus transform [14, 15]. Below we will consider this method and compare it to the structure functions method.

#### Coarse Graining (CG) method

One method which has been applied in several studies is to produce a positive field, called “multifractal random measure”, from a non stationary field such as Temperature and Chlorophyll-a [10, 11, 14]. For that purpose, the gradient modulus of the field *θ* is calculated as follows:

$$\begin{array}{c} \varphi =\sqrt{{\left(\frac{\partial \theta }{\partial x}\right)}^{2}+{\left(\frac{\partial \theta }{\partial y}\right)}^{2}} \end{array}$$ (7)

using at the smallest resolution the discrete transformation:

$$\begin{array}{c} \varphi_{i,j}^{2}={\left(\frac{\theta_{i+1,j}-\theta_{i,j}}{a}\right)}^{2}+{\left(\frac{\theta_{i,j+1}-\theta_{i,j}}{a}\right)}^{2} \end{array}$$ (8)

where *a* is a constant corresponding to grid size and *θ*_*i*,*j*_ is the value of the field *θ* at pixel position (*i*,*j*). This relates a fluctuating field *θ* (a passive scalar) to an intermittent and passive field *φ*. The latter is taken as the multifractal measure at the best resolution *l*_0_. The field *φ*_*l*_ at larger scales *l* ≥ *l*_0_ is then estimated by coarse graining:

$$\begin{array}{c} \varphi_{l}\left(x,y\right)=\frac{1}{a_{l}}\int_{B_{l}\left(x,y\right)}\varphi \left(x_{0},y_{0}\right)dx_{0}dy_{0} \end{array}$$ (9)

This is usually done by taking an image of size 2^*n*^×2^*n*^, and degrading the resolution in *p* steps until scale *l* = 2^*p*^ *l*_0_ (2 ≤ *p* ≤ *n*). At each step, one goes from resolution *l* to 2*l* by taking a local average in a square of 4 values and giving to the larger scale cell this average value. The resolution is degraded recursively. As given by Eq (5), the scale-dependant field has scaling statistics with a scale invariant moment function *K*(*q*), $\langle \varphi_{l}^{q}\rangle \approx l^{-K(q)}$. Experimentally, the function *K*(*q*) is estimated as the regression of $\log\langle \varphi_{l}^{q}\rangle$ versus log(*l*), for each value of *q* (in practice *q* ≥ 0 varies from 0 to 5).

#### Structure Function (SF) method

In fact the application of the gradient modulus method is not necessary to consider the intermittency properties of a 2D field, *θ*, such as temperature and Chlorophyll-a. Let us consider two points *M* and *N* belonging to the field, and their distance *d*(*M*,*N*). The moments ⟨∣*θ*(*M*) − *θ*(*N*)∣^*q*^⟩ versus *d*(*M*,*N*) are considered. This can be estimated directly by taking all couple of points (*M*,*N*) in the 2D domain and discretizing the distance *d*(*M*,*N*) in small intervals. A log-log regression of ⟨∣*θ*(*M*) − *θ*(*N*)∣^*q*^⟩ versus *d*(*M*,*N*) gives the exponent *ζ*_*θ*_, following the law

$$\begin{array}{c} \left\langle |\theta \left(M\right)-\theta \left(N\right){|}^{q}\right\rangle \approx d{\left(M,N\right)}^{\zeta_{\theta }\left(q\right)} \end{array}$$ (10)

where “≈” means scaling relation. In practice, for an image of size *n*×*n*, *M* is chosen among *n*×*n* values and the same for *N*, which corresponds to consider *n* ^4^ couple of points. If *n* = 10^3^, this will provide 10^12^ couple of points, which is usually much too computationally expensive, even for modern computers. It is then necessary to use a numerical method to optimize the computations. *M* and *N* are here randomly taken. The *N*_*p*_ number of couple of points (*N*_*p*_ ≪ *n* ^4^) are taken small enough for a computational realistic time (less than half an hour for each image for a powerful personal computer), and large enough to have converged statistics. The exponent function *ζ*_*θ*_(*q*) is directly estimated from such images using the randomly selected couple of points, *N*_*p*_.

### Tests on 2D stochastic simulation

#### CG Method

In the following, the coarse graining method is tested in 2D with two classical cascade models: the *β* model and the Log-normal model.

#### *β* model 2D cascade

This is one of the first and simplest cascade models to describe the intermittency in turbulence, also called as the black and white model [16]. This model was introduced under this name by Frisch et al. [16], but it has already been discussed by Mandelbrot [17] using a “pulses into pulses”approach originally proposed by Novikov and Stewart [18]. The *β*-model is a discrete multiplicative model. The multiplicative cascade yields a small scale field *ϵ*(*x*) at the smallest scale, as the product

$$\begin{array}{c} \epsilon \left(x\right)=\prod_{i=1}^{n}W_{i,x} \end{array}$$ (11)

of *n* independent realisations *W*_*i*,*x*_ of a random variable *W* (here *x* is the position and *i* is the level in the cascade).

The *β*-model is a binomial model with only 2 possibilities for the value of *W* (0 < *β* < 1):

$$\begin{array}{c} \left\{ \begin{array}{c} \mathrm{Pr}(W=0)=1-\beta \\ \mathrm{Pr}(W=\frac{1}{\beta })=\beta \end{array}\right. \end{array}$$ (12)

We can verify that such field is normalized:

$$\begin{array}{c} \left\langle W\right\rangle =\sum W_{i}\mathrm{Pr}\left(W_{i}\right)=(\frac{1}{\beta })\beta =1 \end{array}$$ (13)

The statistical moments of the random variable *W* are:

$$\begin{array}{c} \left\langle W^{q}\right\rangle =\int W^{q}\mathrm{Pr}\left(W\right)dw=\sum_{i=1}^{n}W_{i}^{q}\mathrm{Pr}\left(W_{i}\right)=\beta^{1-q} \end{array}$$ (14)

The cascade field *ϵ* is built by multiplying *n* independent realisations of *W*. Hence its moments write:

$$\begin{array}{c} \langle \epsilon^{q}\rangle =\langle {\left(\overset{n}{\underset{i=1}{{\prod^{\text{​}}}^{\text{​}}}}W_{i,x}\right)}^{q}\rangle =\overset{n}{\underset{i=1}{{\prod^{\text{​}}}^{\text{​}}}}\langle {\left(W_{i,x}\right)}^{q}\rangle ={\langle W^{q}\rangle }^{n}=\beta^{\left(1-q\right)n} \end{array}$$ (15)

**Fig 1:**
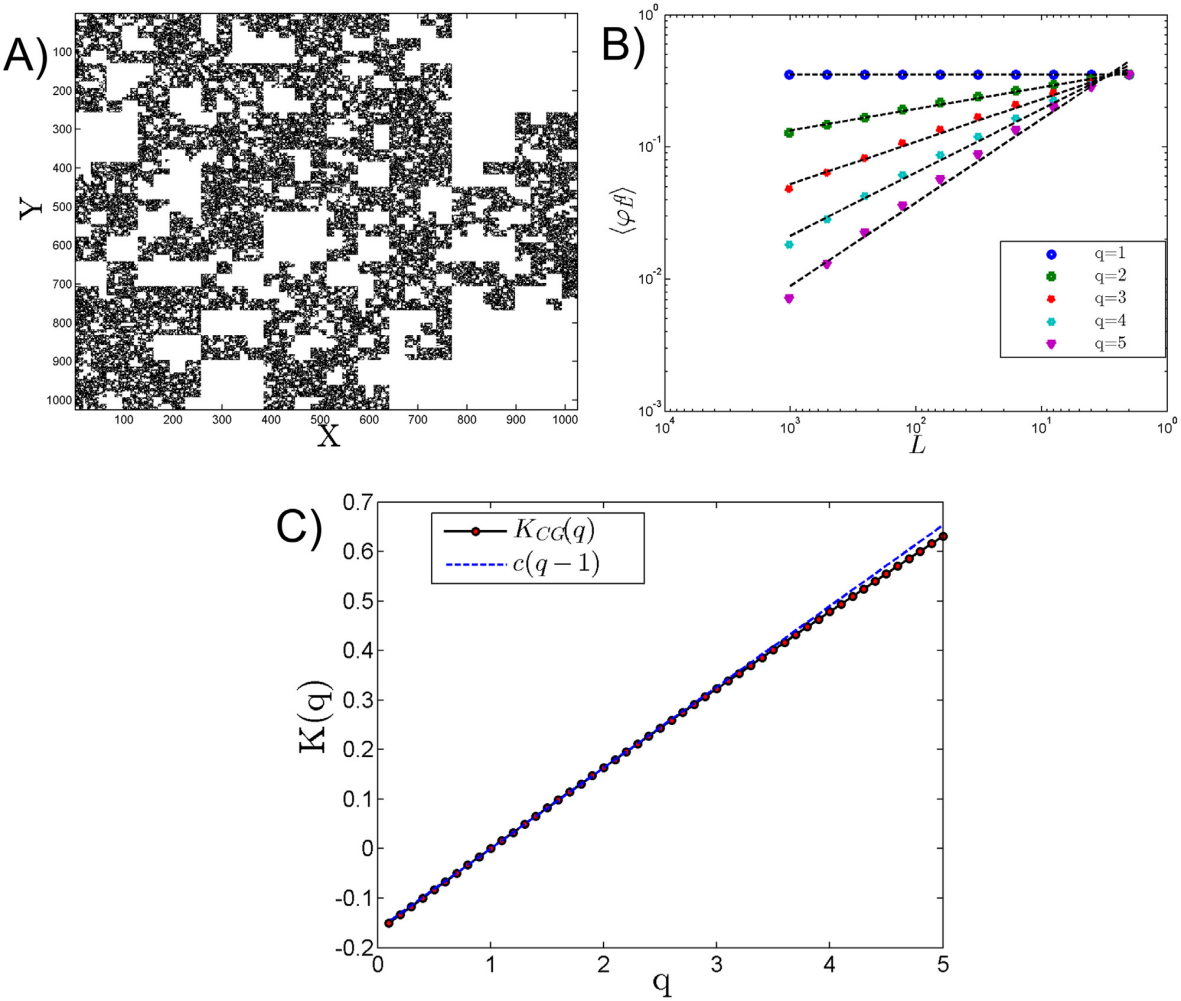
A) simulation of a 2D *β* model with *n* = 2^10^ and *β* = 0.9 (*ϵ* is displayed), B) coarse grained moments for q = 1 to 5 and C) moment scaling function *K*(*q*), where the experimental estimation is shown in dots compared to the theoretical prediction as a dotted line, with *c* = 0.15.

Since each cascade step is associated with a scale ratio of 2 from one scale to the next, we have *λ* = 2^*n*^, where *λ* is the total scale ratio. Hence we have the scaling relation for moments ⟨*ϵ* ^*q*^⟩ = *λ* ^*K*(*q*)^ with *K*(*q*) = *c*(*q* − 1), where *c* = −log_2_ *β* is the co-dimension. Which give rise to Eq (5) by coarse-graining. The scaling moment function *K*(*q*) is linear, and corresponds to a mono-fractal process. A realisation with *n* = 10 and *β* = 0.9 is shown in Fig 1A. Fig 1B shows the CG method applied to this field and Fig 1C, the scaling moment function.

#### Log-normal 2D cascade

The cascade generation for the log-normal model is similar to the *β* model cascade. The only difference is that here we use *W* = *e* ^*g*^, where *g* is Gaussian. As above, the scaling moment function for the dissipation is ⟨*ϵ* ^*q*^⟩ = *λ* ^*K*(*q*)^, where *K*(*q*) = log_2_⟨*W* ^*q*^⟩. To understand the scaling moment function, some basic characteristics of a log-normal random variable are now provided. The moment generating function of a log-normal series (X) of mean *m* and standard deviation *σ* (of logX) is ⟨*X* ^*q*^⟩ = exp(*qm* + *q* ^2^ *σ* ^2^/2). This can be applied to the moment generating function for dissipation:

$$\begin{array}{c} K\left(q\right)=\log_{2}\left\langle W^{q}\right\rangle =\frac{qm+q^{2}\sigma^{2}/2}{\log2} \end{array}$$ (16)

Since we want to have $K(q)=\frac{\mu }{2}\left(q^{2}-q\right)$, where *μ* = *K*(2) is the intermittency parameter, the adequate choice for the discrete log-normal cascade is to take for *g*, a Gaussian random variable of mean $m=\frac{-\mu \log2}{2}$ and variance *σ* ^2^ = *μ*log2.

**Fig 2:**
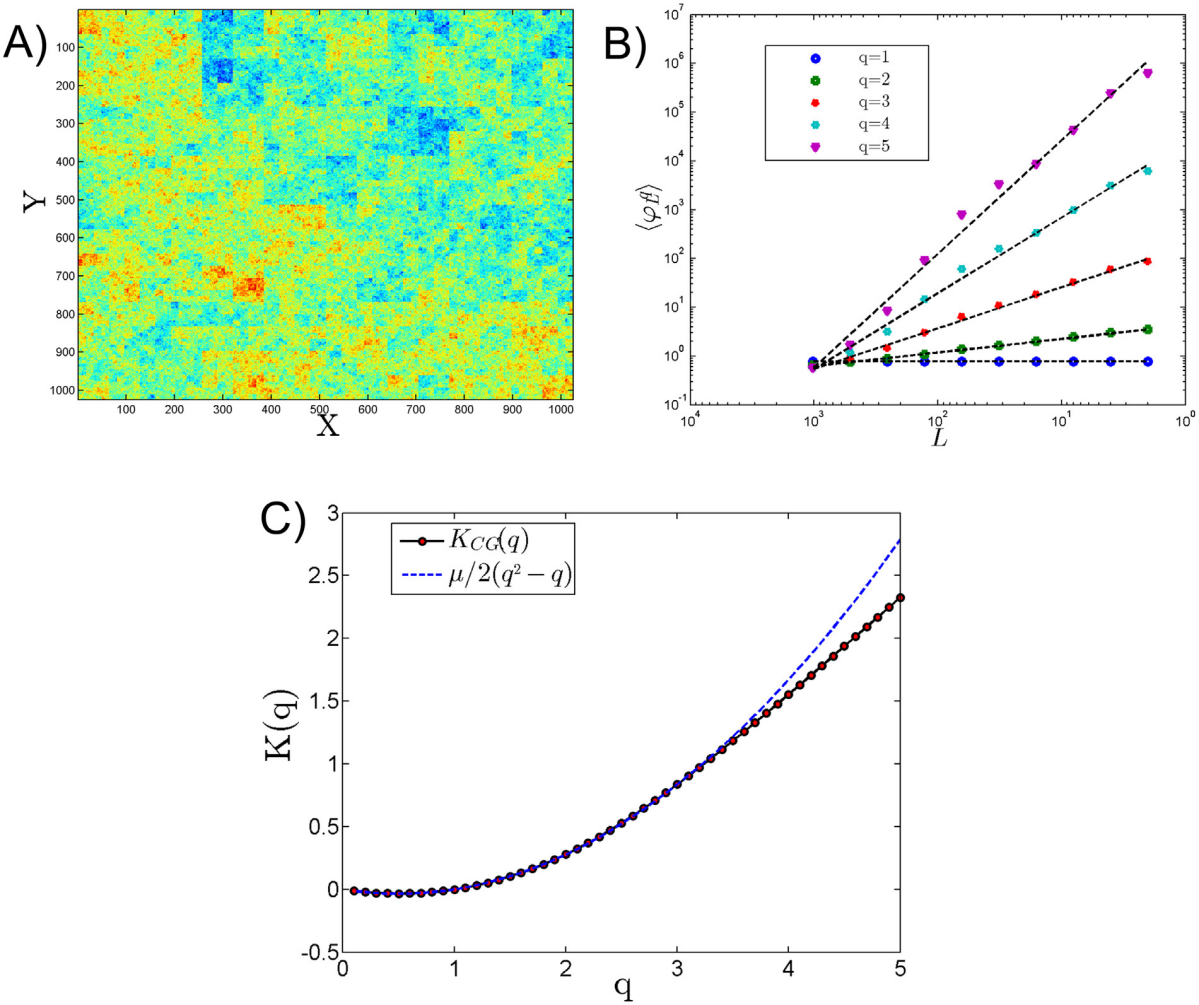
A) Simulation of a 2D log-normal image using a discrete cascade model with *μ* = 0.3, B) coarse grained moments from q = 1 to 5 and C) the corresponding moment scaling function experimentally estimate as dots and theoretical value $K(q)=\frac{\mu }{2}\left(q^{2}-q\right)$ as a dotted line.

A realisation of discrete log-normal cascade has been produced with *n* = 10 and *μ* = 0.3 (Fig 2A). The coarse-gaining method is applied to this image in Fig 2B, and the resulting *K*(*q*) function provided by Eq (16) in Fig 2C. The agreement is excellent until moment of order 3; this is a statistical bound of the estimation of moments which is theoretically predicted [19].

#### SF Method

The proposed structure function method has been validated with a 2D fractional Brownian field with H value varying from 0.1 to 0.9 with an increment of 0.1.

#### Fractional Brownian motion (fBm)

A generalization of Brownian motion, was introduced by Kolmogorov in 1940 [20]. This has been extensively studied by Mandelbrot and his co-workers in 1960s [21] and since then, it is considered as a classical scaling stochastic process for time series analysis. For time series, a *fBm*, denoted by *B*_*H*_(*t*), is a zero-mean Gaussian process with stationary increments characterized by the self-similarity parameter *H*, also known as the Hurst exponent. It possesses the following rescaling property:

$$\begin{array}{c} B_{H}\left(\Lambda t\right)\overset{d}{=}\Lambda^{H}B_{H}\left(t\right),\;\forall \Lambda >0 \end{array}$$ (17)

Where $\overset{d}{=}$ means equality of probability distributions. It leads to linear moment functions using structure functions Eq (6): *ζ*(*q*) = *qH*. This can be done also in 2D. The bi-dimensional isotropic fractional Brownian motion with Hurst parameter H is a centered Gaussian field *B*_*H*_ with an autocorrelation function [22]:

$$\begin{array}{c} \langle B\left(\vec{x}\right)B\left(\vec{y}\right)\rangle \propto ∥\vec{x}{∥}^{2H}+∥\vec{y}{∥}^{2H}-∥\vec{x}-\vec{y}{∥}^{2H};\;0<H<1 \end{array}$$ (18)

where $\vec{x}$, $\vec{y}$ $\in R^{2}$ and ∥ . ∥ is the usual Euclidean norm.

**Fig 3:**
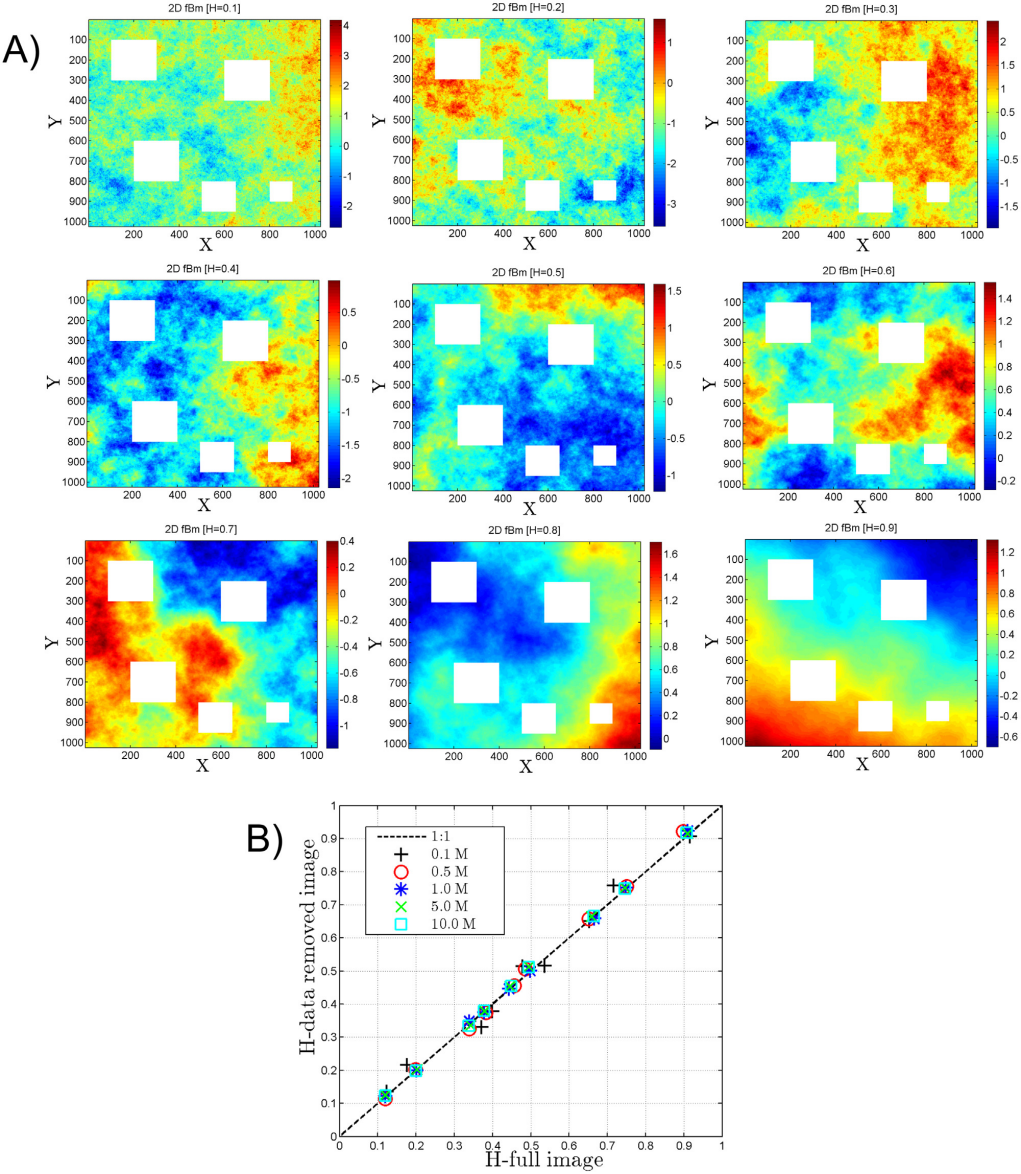
**A) Simulation of 2D fractional Brownian motion for various Hurst exponents (*H* = 0.1, 0.2.., 0.9).** The white rectangles are the space where the data have been removed to test this method for deriving *H* using spatial Structure function method. The 2D structure function was applied to each full image and also to the same image with white rectangles removed, in order to show that this scaling method can be applied to irregular images. B) For each image, comparison of the *H* value estimated using the structure function for the full image and for images with missing values.

In the present study we simulated 2D fractional Brownian field for various *H* values (*H* = 0.1, 0.2.., 0.9) using an algorithm and code described in recent works [23, 24] (Fig 3A). These images are analysed using 2D SF method for various randomly selected data (*N*_*p*_ = 0.1 million, 0.5 million, 1.0 million, 5.0 million and 10.0 million). The scaling moment function has been derived for each image for different iteration number. *H* has been derived from the moment scaling function using *H* = *ζ*(1). Since satellite images often have missing values due to cloud coverage, we have also applied the SF approach to irregular images, where some part of the image have been removed. Fig 3A shows some simulations for various values of H and Fig 3B compares H estimations for full images and for images with some rectangles removed. This is tested for various values of *N*_*p*_. We see that for *N*_*p*_ = 10^6^ the method works very well (with an error of 3.88%) even when there are missing values, and the estimated exponents are very precise. In the following we thus choose *N*_*p*_ = 10^6^, since it is computationally reasonable and provide converged statistics for scaling exponents. To estimate the standard deviation of the estimated values with respect to full image and percentage of missing values will need a systematic study, which will be the topic of a future work.

## Comparison of the CG and SF methods

Two recent studies have proposed to analyse the scaling of satellite images by applying first a gradient modulus approach [12, 14], in order to have a positive intermittent field, and then appying the CG method. The *K*_*CG*_(*q*) exponent function is retrieved and the authors assume that:

$$\begin{array}{c} K_{CG}\left(q\right)=qH-\zeta \left(q\right) \end{array}$$ (19)

where *ζ*(*q*) is the scaling exponent characterizing the Chl-a or SST fluctuations. Based on one simulated image, and two real satellite images we compare this latter approach with the one proposed here. We extract *K*_*CG*_(*q*) as described above, and we directly estimate *ζ*(*q*) using the 2D structure functions. We then compute *K*_*CG*_(*q*) + *ζ*(*q*): if Eq 19 is correct this should be linear (= *qH* = *qζ*(1)).

### Multifractal field from cascade and fractional integration

**Fig 4:**
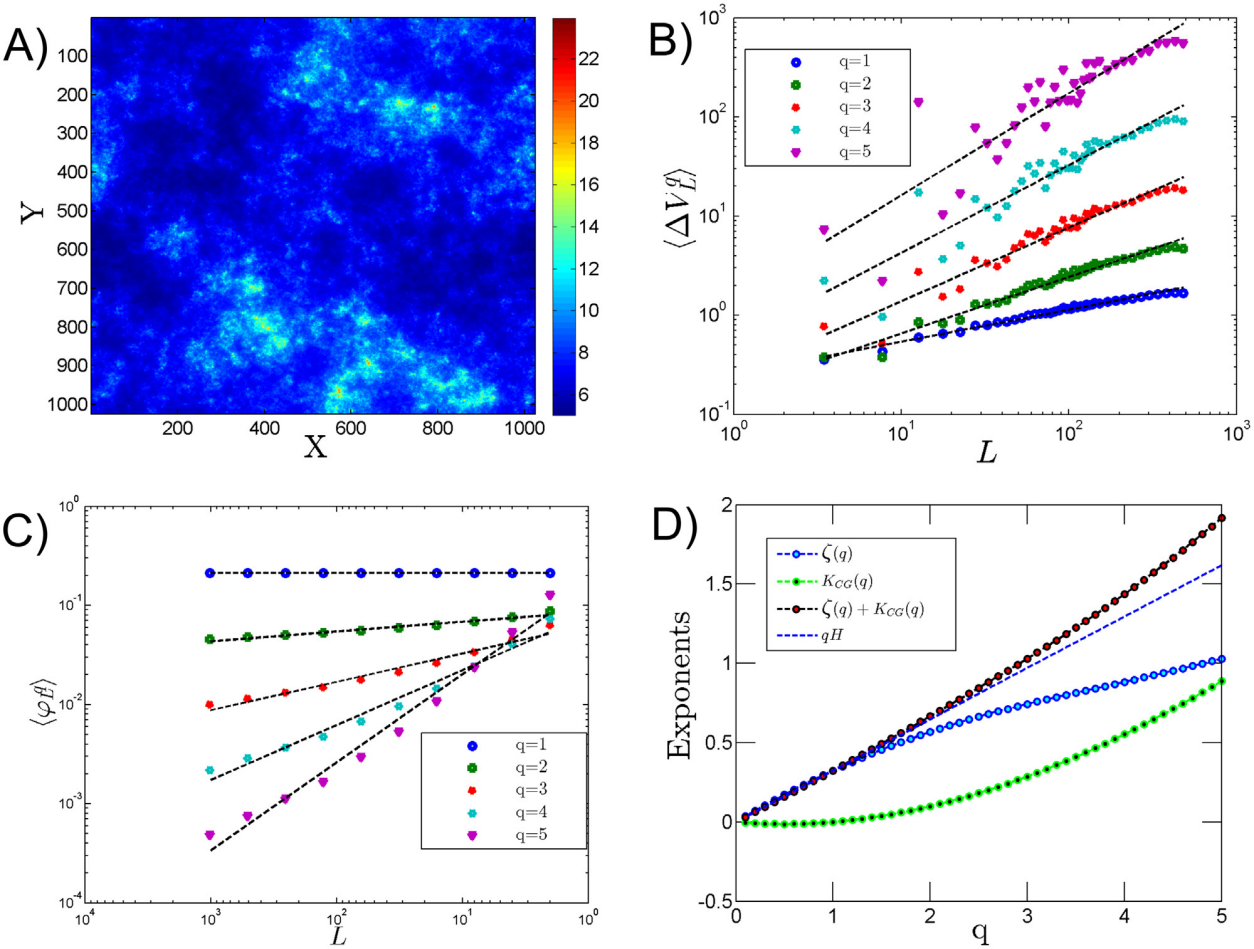
**A) Simulation of a 2D log-normal multifractal image with *H* = 0.35 and *μ* = 0.1.** B) Scaling of the SF; C) Scaling analysis when gradient modulus is applied on the image shown in A; D) representation of different exponents.

We first test Eq 19 using a multifractal simulation done by performing a cascade and then a fractional integration [25]. As done in Lovejoy et al.[15], we simulate a 2D log-normal multifractal image with *H* = 0.35 and *μ* = 0.1 [26] (Fig 4A). The SF is directly applied to the image itself and the CG is applied to its gradient modulus. The scaling moment spectrum is derived for each method (Fig 4B and 4C). The moment scaling functions for both SF and CG are derived for various moments from 0.1 to 5 with an interval of 0.1. *K*_*CG*_(*q*) is non-linear and *ζ*(*q*) is almost linear and Eq 19 is not verified: *K*_*CG*_(*q*) + *ζ*(*q*) is close to *qH* for *q* ≤ 2 but for larger moments it is no more the case (Fig 4D).

### Scaling analysis on a Chl-a image of MODIS aqua

**Fig 5:**
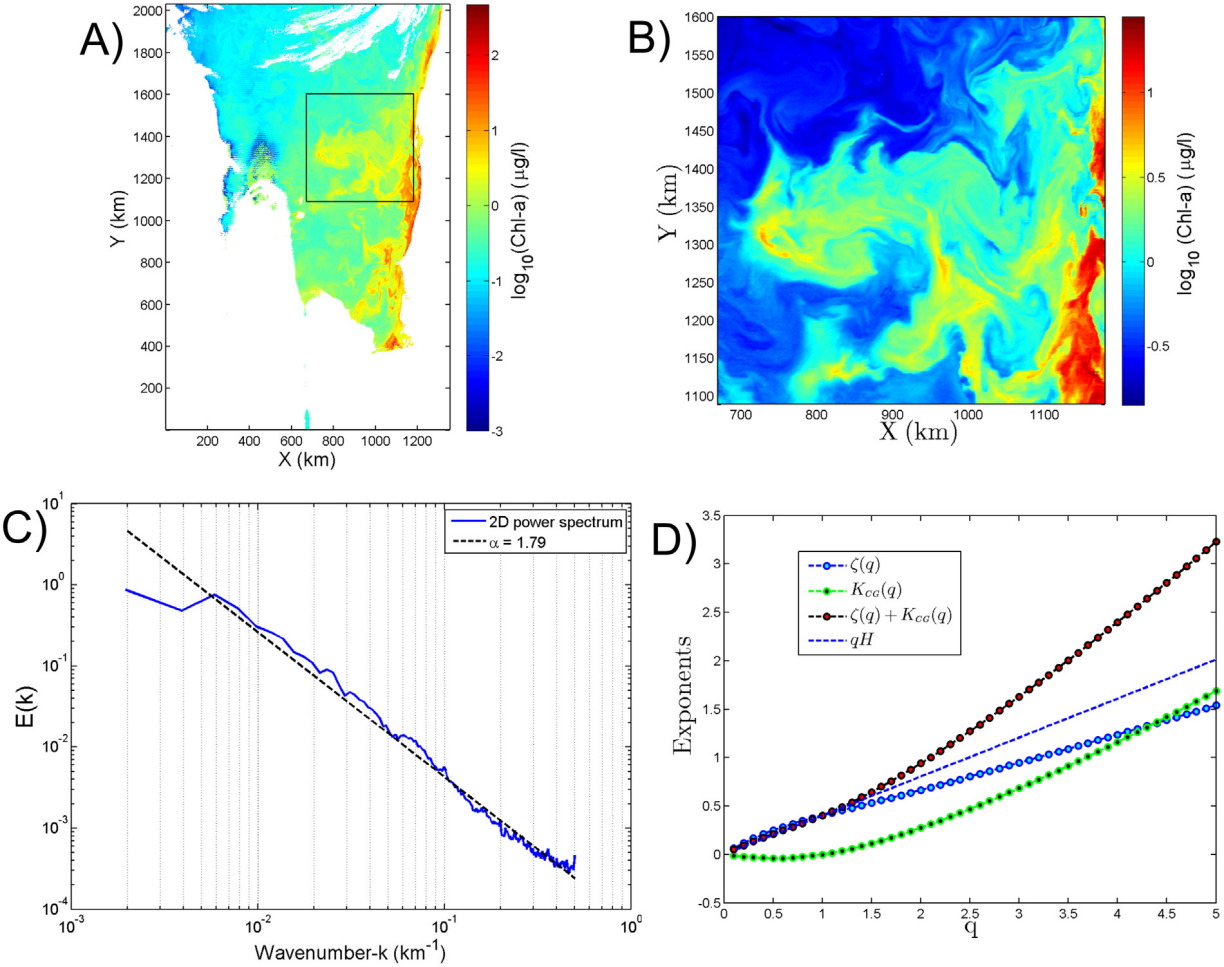
**A) Chl-a image from MODIS Aqua from the Mauritanian coast sampled on 11 March 2003; the square indicates the 512 × 512 pixel of cloud free image chosen for the analysis of Chl-a.** B) Gradient modulus estimated for the latter square image. C) Power-spectrum of the Chl-a image showing a scaling exponent *α* = 1.79. D) Moment scaling function for the square image, using the CG and SF methods. Eq 19 is tested and found not to be correct for *q* ≥ 1.7.

The standard MODIS Chl-a imagery available from the Goddard Space Flight Centre is produced via *OC*3*M* algorithm [27] has been used for the present study. The *OC*3*M* algorithm is a fourth order polynomial equation and applies the maximum ratio of the remote sensing reflectance at 443 *nm* (blue) to 550 *nm* (green) or 490 *nm* (blue) to 550 *nm* (green). These proposed methods have been applied to real images of Chl-a from the Mauritanian coast sampled on 11-March-2003 (Fig 5A). A cloud free image (512 × 512 pixels) has been extracted for the analysis (square region marked in Fig 5A). The gradient modulus of Chl-a (Δ Chl-a) has been derived from the Chl-a image (Fig 5B). This gradient modulus generates a positive field, the CG method is adopted for analysing this positive field. The SF method has been applied directly to the Chl-a image. We have chosen the random picking method tested in section 2 with *N*_*p*_ = 10^6^ couple of points. Here also we could observe the power-law behaviour of the SF. The radially summed power-spectra of the Chl-a image has been derived for the cloud free part of the image (512 × 512 pixel). The derived spectral exponent *α* for the radially summed image is 1.79 (Fig 5C). The constants derived for the Chl-a image are shown in Table 1. The *ζ*(*q*) derived for the Chl-a image also follows a non-linear convex curve showing intermittency in the spatial distribution of Chl-a (Fig 5C). Fig 5D shows that Eq 19 is not verified for *q* ≥ 1.7.

**Table 1:** The exponents (*H* and *α*) derived for Chl-a and SST images for Mauritanian region. The Hurst exponent *H* derived through *SF* (*H* = *ζ*(1)).

Region	Sampling date	Parameter	*H*	*α*
Mauritanian Coast	11-Mar-2003	Chl-a	0.37	1.79
Mauritanian Coast	11-Mar-2003	SST	0.41	1.80

### Scaling analysis of MODIS SST

**Fig 6:**
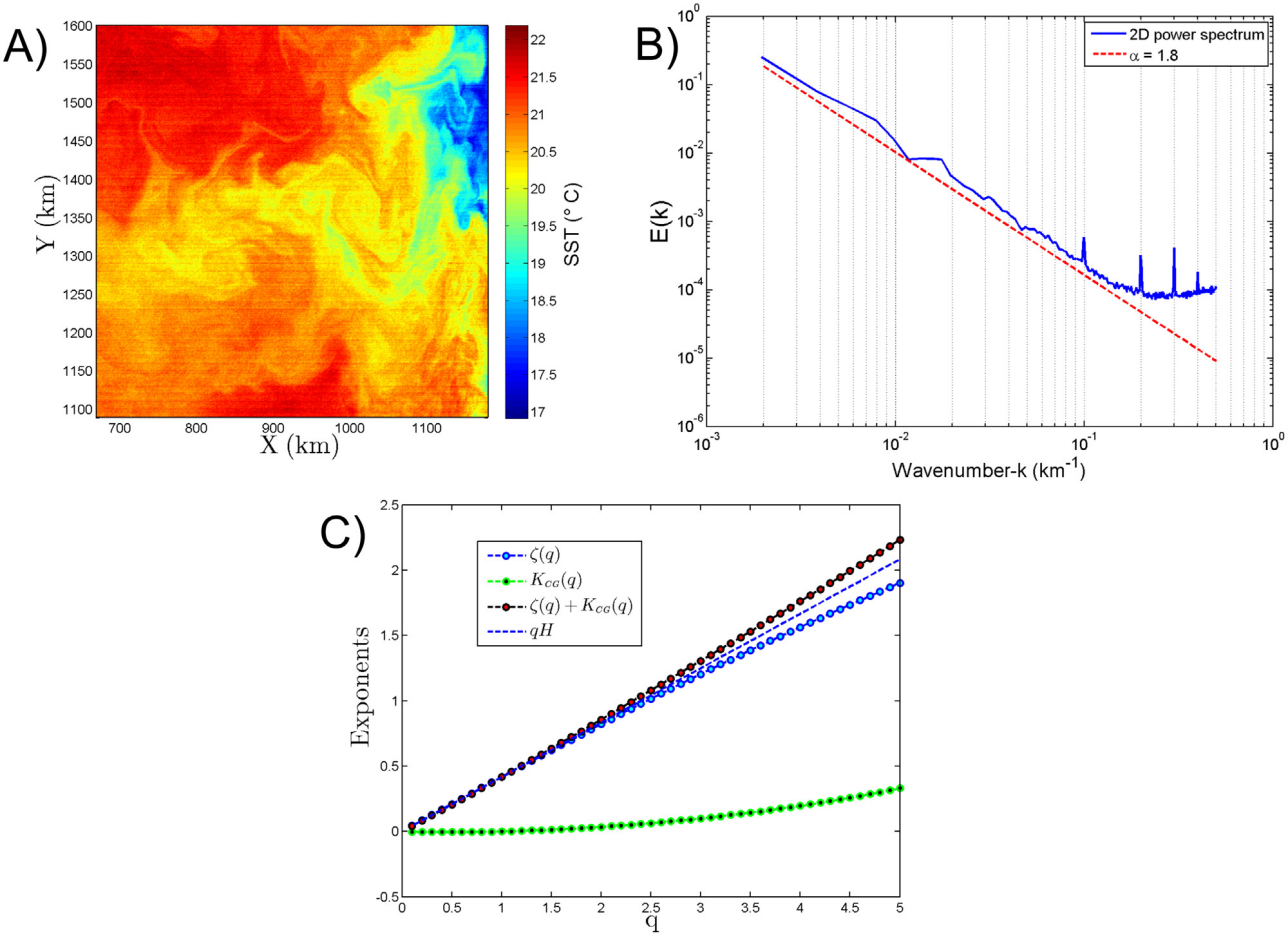
**A) SST image from MODIS from the Mauritanian coast sampled on 11 March 2003.** B) 2D power-spectrum of the image showing a scaling exponent *α* = 1.80. C) Moment scaling function, using the CG and SF method. Eq 19 is approximately valid, coming from the fact the *K*_*CG*_(*q*) is very small, corresponding to a very regular field.

These proposed methods (CG and SF) have also been applied to an image of SST sampled simultaneously with Chl-a from the Mauritanian coast on 11-March-2003. A cloud free image (512 × 512 pixels) has been extracted for the analysis (Fig 6A). The 2D power-spectra of the SST image has been derived. It follows a power-law behaviour with a spectral slope *α* = 1.8 (Fig 6B) with some noise observed at smaller scales. Similarly to Chl-a, the gradient modulus of the SST has been derived and CG method applied. Here also the scaling moment function derived for the SST follows a non-linear concave curve showing the spatial intermittent characteristics of SST (Fig 6C). However its small value shows that this field is not very intermittent. The proposed SF method has been directly applied to the SST image. The moment scaling function of SST image derived follows a non-linear convex shape, showing the intermittent characteristics of the spatial distribution of the SST (Fig 6C). The H derived through the SF method is *H* = 0.41 (*H* = *ζ*(1)). The constants derived for the SST image are shown in Table 1. Here we also can see that these two exponents fall away from the typical linear *qH* line for *q* ≥ 1.5.

The values of the spectral exponent *α* derived here for the Chl-a and SST satellite images are in good agreement with the range of *α* values derived from in situ measurements of fluorescence (as a proxy of Chl-a) and temperature [28]. The estimated values of the scaling parameter *H* for Chl-a and SST show also very good agreement with previous studies [28–30]. Concerning the *μ* value, let us note that this intermittency parameter could be more sensitive to the local conditions. It can be estimated using structure functions as *μ* = *K*_*CG*_(2) = 2*ζ*(1) − *ζ*(2). For Chl-a and SST we obtain here 0.13 and 0.012 respectively. For Chl-a, this value is larger than the one estimated from Eulerian time series, ranging from 0.065 to 0.074 [28, 31]. On the other hand, the SST field considered here seems smoother than found in other studies since *μ* = *K*_*CG*_(2) in other published studies range from 0.05 to 0.19 [28–32].

## Discussion: the role of signs

**Fig 7:**
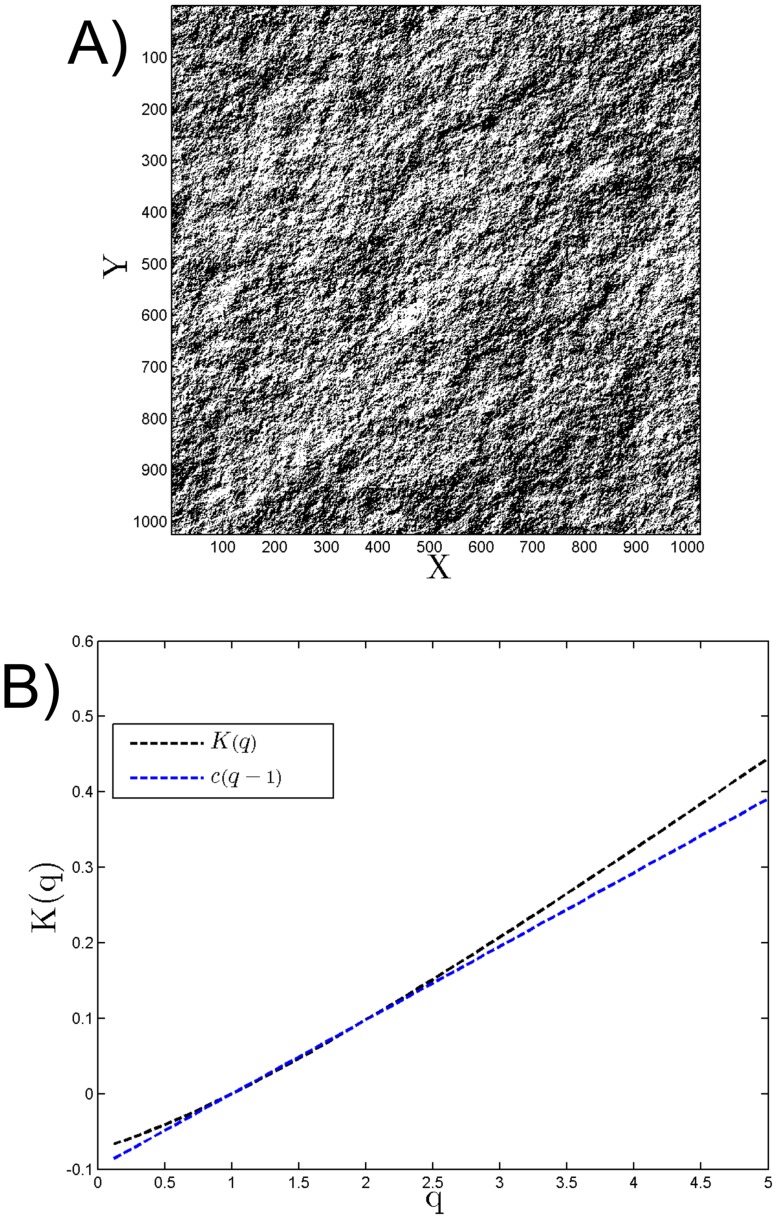
The gradient sign information of the 2D fBm derived for *H* = 0.6 in A) and its moment scaling function in B).

We have considered here the scaling exponent *K*_*CG*_(*q*) obtained by coarse-graining a positive field, and the exponent *ζ*(*q*) obtained directly through structure functions. We found, using a simulation and two satellite images, that Eq 19 is not verified, an equation assuming that the gradient modulus applied to a non-stationary field retrieves the basic scaling information. In fact, such relation is not verified because the local sign contains information; when performing a gradient modulus, the sign information is lost. We check this hypothesis here by considering a fBm simulation with *H* = 0.6. We estimated a sign information from the 2D fBm simulation as follows. We computed the two components of the gradient (in the *x* and *y* directions) and took the sum of the two terms. If this sum is positive, we choose to consider a sign information as 1 and 0 if the sum is negative. This way the sign information of the gradient is transformed into a matrix containing only 0 and 1 values. The figure obtained (Fig 7A) does not seem to be a noise; to check this we consider its scaling by using a coarse-graining (Fig 7B). We obtain a scaling law of the form *μ*(*q* − 1) with *μ* = 0.09. This is similar with *β*-model and shows that the sign information has a structure; such structure is lost when performing a modulus and we can assume the same property for real images: such analysis is left for future studies.

## Conclusion

We have considered here several methods to estimate the scaling properties of ocean colour images, in relation with turbulence. We have first recalled data analysis methods, mainly coarse graining after taking the gradient modulus, and 2D structure functions. Similar to many atmospheric processes, oceanic processes are also governed by complex turbulent processes. These processes cannot be fully characterised by a single scaling exponent such as *α*. Additional multifractal parameters are required to fully characterise these multi-scaling properties. Even though the CG method is successful in many applications, it suffers from several approximations that can add some uncertainties in the estimation of multifractal parameters. In this context, we highlighted here an alternative tool such as 2D structure function to overcome the approximations related to the CG method. This method of 2D structure functions has rarely been documented and studied for geophysical image analysis due to computational complexity constraints. We have obtained several results in this framework:

- Since the structure function approach needs to consider *n* ^4^ couple of points, where *n* is the linear size (in pixels) of an image, it is too much computer time consuming. We shown using fBm simulations that taking 10^6^ couple of points randomly is enough for an adequate estimation of the structure function scaling exponents. We showed also that this method works for images with missing data, an important aspect since many real images have missing pixels due to cloud coverage.
- We compared the Coarse graining scaling exponent *K*_*CG*_(*q*) from the gradient modulus, to *qH* − *ζ*(*q*), and found that such relation is not verified, indicating that the gradient modulus looses information (the signs have a scaling structure) and hence this method cannot be safely used instead of SF.
- We considered two images from MODIS Aqua (Chl-a and SST) and showed on these examples that scaling approach using SF and *N*_*p*_ = 10^6^ couple of points is adequate; we also showed that the spectral exponent for these examples is close to 5/3 characteristic of passive scalar fully developed turbulence. Such 2D multifractal property of Chl-a and SST is a 2D generalistic of previous results obtained for time series [28, 31, 32].
- Since Chl-a and SST are not conservative, Chl-a can be influenced by biological activities and SST can be influenced by the surface heat flux. These biological and physical processes can have influence on the scaling exponents. These two parameters may show different scaling properties for in situ measurements as shown in other studies [28, 31]. The spectral exponent *α* derived for Chl-a and SST satellite images are in good agreement with the in situ measurements of fluorescence by Chl-a and temperature [28, 31, 32].
- The present paper compared CG and SF methods on a real image. We have considered here the question of missing data on a synthetic fBm field; the same has been done on real images and it was confirmed that the method is also providing the same scaling exponents for real images (not shown here).

Let us note that this method can also be applied to the 2D velocity field obtained from altimeter data, since the velocity can also be intermittent and scaling. As a perspective, in a following work, we will use the SF method with *N*_*p*_ = 10^6^ couple of points, to estimate the *ζ*(*q*) function, fit with the data using a log-normal approximation with 2 parameters (*H* = *ζ*(1) and *μ* = 2*H* − *ζ*(2)) and consider the values of these parameters in several locations (open ocean, coastal waters, upwelling region, etc.). For that purpose, several images collected over different oceanic regions characterised by contrasted biological and physical environment will have to be studied.
